# Predictors of Trauma Exposure and Trauma Diagnoses for Children with Autism and Developmental Disorders Served in a Community Mental Health Clinic

**DOI:** 10.1007/s10803-019-04331-3

**Published:** 2019-12-14

**Authors:** John D. Hoch, Adriana M. Youssef

**Affiliations:** 1 Fraser, 3333 University Ave SE, Minneapolis, MN 55414 USA; 2 Institute for Translational Research in Children’s Mental Health, University of Minnesota, 1100 Washington Avenue South, Minneapolis, MN USA

**Keywords:** Autism spectrum disorders, Developmental disabilities, Trauma, Logistic regression, Potentially traumatic experiences

## Abstract

Exposure to potentially traumatic events (PTEs), and trauma related diagnoses are poorly understood in autism spectrum disorders (ASD) and developmental disabilities (DD). The current study examined N = 7695 cases seen by a community mental health provider to compare exposure to PTEs and trauma-related diagnoses between children with ASD, children with DD, and children with other mental health diagnoses (e.g., depression). Predictors included demographics, exposure to negative life events, living situations, and subscales of the strengths and difficulties questionnaire (SDQ). Logistic regressions showed that diagnostic group, number and type of negative life events and locations lived, and SDQ subscale scores predicted trauma reports and trauma diagnoses. The findings suggest screener questions that may be useful across diagnostic groups.

In the general population, prevalence rates of childhood exposure to potentially traumatic events (PTEs) and rates of trauma and stressor-related disorders have been well documented. This research base has guided the development of trauma-focused evidence-based interventions (Copeland et al. 2007; Leenarts et al. 2013). However, very little is known regarding trauma exposures and traumatic stress effects in children with autism and developmental disabilities. Of interest to the current study are two groups of children for which social, behavioral, communication, and cognitive difficulties may make them particularly at risk for PTEs: (1) those with a developmental disability (DD), such as intellectual disabilities and global developmental delays, and (2) those with autism spectrum disorder (ASD). While individuals with a DD have impairments in cognitive functioning and adaptive behaviors, those with ASD may have additional unique cognitive, communication, behavioral, and social features which may put these individuals at an even greater risk for experiencing PTEs. The communication difficulties characteristic of ASD may also make it more difficult to obtain reports of trauma and to ascertain traumatic stress effects on behavior than those with other disabilities. As a result, many children with ASD may be under-identified for trauma exposure and under-served for treatments to address the trauma compared to children with DD or to those with other mental health diagnoses such as depression or anxiety. For these groups of children it is important to understand how they compare to their peers in the types of PTEs that may lead to caregivers reporting trauma, and to clinicians diagnosing trauma related disorders in comparison to other clinical samples.

In general, PTEs are associated with significantly increased rates of physical, mental, and psychosocial problems in childhood (Felitti et al. 1998; Shonkoff et al. 2012). One potential outcome of childhood exposure to PTEs is the development of trauma and stressor-related symptoms such as avoidance of stimuli associated with the event, severe anxiety reactions triggered by stimuli related to the event, or nightmares. The diagnostic and statistical manual of mental disorders, fifth edition (DSM-5) category of trauma and stressor-related disorders includes several specific disorders that all require exposure to a PTE and reactions to this event (American Psychiatric Association 2013). The most commonly discussed of these disorders is Post-Traumatic Stress Disorder (PTSD) which is seen in 0.1% of typically-developing children in the population aged 9–16 years, with another 0.9% showing subclinical symptom levels (Copeland et al. 2007). Other stress-related disorders include reactive attachment disorder and acute stress disorder. Childhood exposure to trauma and resulting psychopathology may contribute to poor overall well-being into adulthood, and even early mortality (Felitti et al. 1998; Shonkoff et al. 2012). Given the paucity of research conducted examining trauma in children with DD and ASD, along with the growing numbers of children diagnosed with ASD each year (Xu et al. 2018), it is critical to examine prevalence rates and potential risk factors of exposure to PTEs and trauma and stressor-related diagnoses in children with these disabilities. This work will help inform future research in developing evidence-based screening and assessment measures along with treatments to address trauma in individuals with a disability.

## Literature Review

### PTE Exposure

Some research suggests children with special needs identified by diagnostic categories such as ASD or DD, or by special education service categories may experience trauma at an alarmingly higher rate than their same age peers (Berg et al. 2016; Brenner et al. 2018; Hall-Lande et al. 2015; Mandell et al. 2005; Sullivan and Knutson 2000). In addition to experiencing trauma, emerging evidence suggests that children with some disabilities may also be more vulnerable to PTEs (Spencer et al. 2005; Sullivan and Knutson 2000). Kerns et al. (2015) conducted a review of the literature specific to children with ASD and discuss how psychosocial processes may put those with ASD specifically at risk for trauma exposure. Social skills deficits, high levels of bullying and peer victimization, and dependency on caregivers for an extended period of time may make this population a target for abuse (Little 2001, 2002; Focht-New et al. 2008; Rose et al. 2011; Sabornie 1994; Sreckovic et al. 2014; van Roekel et al. 2010; Zeedyk et al. 2014). Kerns et al. (2015) also suggest that individuals with autism may be more susceptible to perceive non-threatening stimuli as threatening, due to misunderstanding of social cues and situations. Sensory sensitivities such as heightened sensitivity to noise and other stimuli (Wiggins et al. 2009) may also lead to stimuli that are perceived as non-threatening by most children to be seen as threatening by children with ASD.

Empirical studies of ASD and DD, and related educational categories have indeed documented higher levels of PTE exposure than non-diagnosed peers. For example, children with learning disabilities in an educational program have been found to experience more negative life events than those in general education (Hatton and Emerson 2004). In a population-based study of elementary age children, those receiving special educational services for an intellectual disability made up 25% of police records indicating maltreatment, with neglect being the most predominate form of abuse followed by physical, emotional, and sexual abuse in that order (Sullivan and Knutson 2000). In a study comparing those with ASD to typically-developing peers in the general population, those with ASD were found to be twice as likely to experience 4 or more adverse childhood experiences (Berg et al. 2016). To date, only one study has compared children with ASD and DD to typically-developing peers, all of which were sampled from a database of families involved in the child protection system. This study found that those with ASD and DD were more likely to receive services from child protection than children with no disabilities (Hall-Lande et al. 2015).

Among typically-developing populations, exposure to PTEs has been linked to the development of psychopathology including trauma and stressor-related disorders (Davis and Siegel 2000 for review). Several factors may put individuals with a disability exposed to trauma at risk for developing psychopathology. Children with ASD and DD may experience a lack of social support and less engagement with peers to help combat the negative effects of trauma (Bauminger and Kasari 2000; Chamberlain et al. 2007; Lasgaard et al. 2010). Regarding individuals with ASD specifically, Haruvi-Lamdan et al. (2018) suggest that cognitive traits associated with ASD such as high levels of rumination on past events and lack of cognitive flexibility could increase the subjective impact of stressful events and lead to higher levels of stressor-related disorders. Based on a review of the neuropsychobiological literature, Kerns et al. (2015) suggest neurobiological processes that may increase the risks of PTSD in ASD. The authors discuss neurobiological evidence showing similarities in the functional connectivity and structure of the amygdala and prefrontal cortex, areas important in the regulation of emotions, between people diagnosed with ASD and those with trauma histories, PTSD, and other trauma-related diagnoses (Grant et al. 2011; Mazefsky et al. 2013; Williams et al. 2006). Similarly, limbic–hypothalamic–pituitary–adrenal (LHPA) axis disruption has been documented in ASD, primarily in studies of cortisol response to stressors and disrupted daily rhythms of cortisol variation that may lead to increased sensitivity to stress or may indicate a stress system disruption (Corbett et al. 2008. 2009).

Several studies have demonstrated associations between exposure to PTEs and problems in emotional, behavioral, adaptive functioning across the lifespan in those with a DD and ASD (Kerns et al. 2015; Wigham et al. 2011 for review). However, little work has been done specifically examining trauma and stressor-related disorders among these children. Brenner et al. (2018) compared parent reports of specific PTSD associated symptoms within a sample of children with ASD who had been served in an inpatient psychiatric program across multiple sites in the United States. The study found that 28% of the sample reported some form of abuse or neglect and 2.6% had diagnoses of both ASD and PTSD. The most common forms of abuse were physical, emotional, and sexual abuse (reported in 13%, 12% and 8% of the sample respectively). Another study of children with ASD receiving services within an outpatient clinic in Turkey, found prevalence rates of PTSD in those with abuse histories as high as 67% (Mehtar and Mukaddes 2011). While these studies suggest relatively high levels of PTSD in ASD, other studies suggest an under-diagnosis of this disorder. For example, in their investigation of high school youth with ASD who had experienced trauma, Taylor and Gotham (2016) found that none of the sample met criteria for PTSD, whereas 25% met criteria for a mood disorder and 25% met criteria for an anxiety disorder. Similarly, in their examination of psychiatric comorbidity in children aged 6–12 years with a diagnosis of Pervasive Developmental Disorder-Not Otherwise Specified (a diagnostic category from the DSM-IV now subsumed into ASD in the DSM-5), a significant portion (80.9%) had at least one comorbid diagnosis and yet none of this sample had an identified diagnosis of PTSD (de Bruin et al. 2007). These contrary findings underscore the need to examine demographic and contextual-level factors which may aid clinicians in the proper identification of those at-risk for trauma and stressor-related disorders. Indeed, studies in typically-developing children have identified demographic and contextual-level factors such sex, age, family history of mental health problems, and socioeconomics as being key sources of risk for the development of PTSD (see Davis and Siegel 2000 for review).

Much of the empirical evidence has not utilized clinical diagnosis of ASD or DD using standardized measures. Rather, much of this research has used school records, receipt of special education services, and/or parent report of symptoms to define children with “special needs” making it difficult to ascertain whether children with DD or ASD might be more vulnerable than typically-developing children to trauma exposure and its effects. Additionally, updates to the DSM-5 have been made to the diagnostic criteria for ASD and PTSD which have likely influenced the prevalence rates of these diagnoses. For instance, a diagnosis of ASD in the DSM-5 now includes those individuals who had previously been given other childhood disorders such as pervasive developmental disorder-not otherwise specified (PDD-NOS) or Asperger’s Disorder. Changes to the PTSD criteria involved adding three new symptoms, revising some symptoms to clarify expression, creating a separate diagnostic category for children younger than 6 years, and removing the criteria that the individual had to have experienced immediate intense fear, helplessness, or horror after exposure to trauma (American Psychiatric Association 2013). These changes necessitate a re-examination of trauma prevalence and trauma-related disorders in the ASD group as compared to children with a clinical diagnosis of DD and those with other mental health diagnoses, such as depression and anxiety. As much of the empirical evidence has only examined PTSD in relation to disabilities, an examination of other stressor and trauma-related disorders as detailed in the DSM-5, such as Reactive Attachment, Acute Stress, Disinhibited Social Engagement, and Adjustment Disorders, are also needed.

### Literature Summary

In summary, there is some information available to support the idea that both exposure to stressors and PTEs are seen at high levels in people with ASD and DD’s. Despite this, trauma and stressor-related disorders may be under-diagnosed in these individuals. Studies that compare cohorts of children with ASD to those with other disorders are lacking, as well as studies of population-based cohorts in community mental health settings where children with developmental delays typically receive services. There remains a lack of studies using validated measures or clinical assessment of diagnoses of ASD and DD. It is also unclear what types of negative life experiences and other demographic factors might be significant for detecting trauma and trauma-related disorders in populations being served within community mental health settings. Clinically, there are challenges in identifying PTEs in ASD and DD populations, and then detecting the reactions to these PTEs against a background of communication and social deficits.

### Present Study

#### Objectives

The current study uses a large sample (N = 7695) of children and adolescents seen at an urban Midwestern provider best known in the community for services for autism and developmental disorders, that also provides services across the range of mental health disorders. Because of the availability of large numbers of comparison participants who are also in need of clinical intervention, but who do not have ASD or DDs we are able to compare those with ASD diagnoses or DD diagnoses to children seen at the same clinic with a broad range of other mental health disorders. Data on trauma reports (PTEs) and a wide range of Negative Life Events (NLEs) were collected during comprehensive clinical diagnostic interviews. We conceptualize the trauma reported by parents as PTEs for the purpose of this study as they include categories of events that are typically considered likely to result in trauma reactions (e.g. physical abuse). We consider other NLE’s (e.g. frequent moves), as possible predictors of trauma because they are often things that may be associated with increased risk for trauma exposure but that are not typically triggers for trauma in themselves. The current study sample spans a larger age range than previous studies allowing the effect of age to be examined statistically. Because clinical diagnoses are expected to be rare in the age range examined here, the study also examines how caregiver report of trauma differs between these groups. We are seeking specific predictors from the types of NLEs, demographic information, and subscales and scores from a broad psychological screening tool, the strengths and difficulties questionnaire (Goodman 2001) that may be useful in clinical practice.

#### Hypotheses

Based on the current literature it is difficult to make informed predictions about whether children with ASD will show higher or lower levels of trauma report, and trauma related diagnoses compared to those with DD and those in the comparison group of children with other mental health diagnoses. Associations with SDQ subscales and demographic variables are also exploratory. It is unclear whether specific subscales, or demographic variables such as race and primary language may be associated with trauma, or whether these relationships will be different for the diagnostic groups in the study. Across all diagnostic groups, it is hypothesized that more negative life experiences will be associated with a greater likelihood of trauma reports.

#### Research Questions

Specific research questions include: Does overall number of negative life experiences, or specific kinds of negative life experiences change the likelihood of trauma reports from parents, or diagnoses of trauma stressor-related diagnoses? Are the types and numbers of these experiences different between children with ASD, compared to those with DD or other mental health disorders? Are there SDQ scales or other demographic variables that might help target prevention and trauma screening in these populations?

#### Clinical Significance

Implicit in this research is a search for potential screening tools or questions that may provide ways to identify groups at high risk for trauma exposure and diagnoses while asking questions of parents that are less sensitive and easier to recall than direct queries about trauma exposure. This research may also be important in developing or adapting interventions for children with ASD or DD, particularly if specific types of NLE’s are differentially identified for these children.

## Methods

### Procedures

A data sharing agreement was reviewed by the Institutional Review Board at the University of Minnesota (IRB#1609S94221) to allow sharing of de-identified data from Fraser, a mental health provider located in Minneapolis, Minnesota, a major metropolitan area in the Midwest. Data included in this study spanned the data range from August of 2013 to February of 2018.

The provider conducted diagnostic assessments and evaluations for autism and other mental health disorders across the lifespan. Full evaluations were typically conducted with a psychologist and a mental health professional (typically a master’s level clinician such as Licensed Clinical Counselors, or Licensed Social Workers) working as a team in a three hour clinic visit. The psychologist typically measured cognitive ability using standard IQ tests and conducted child observations and used child measures specific to the diagnostic question (e.g. Autism Diagnostic Observation Schedule). The mental health professional conducted structured interviews with the child’s caregivers and completed measures of adaptive functioning (e.g. the Vineland Adaptive Behavior Scales; Sparrow et al. 2016). Parent rating scales such as the Child Behavior Checklist (Achenbach 1994), and the Autism Spectrum Rating Scales (Goldstein and Naglieri 2010) were also frequently completed by parents with the mental health professional. See Table 1 for a list of the most commonly used measures from assessments.

**Table 1:** List of measures used in evaluations and diagnostic assessments

Test name	Distinct participants
Strengths & Difficulties (SDQ)	2868
Child&Adol. Service Intensity (CASII)	1904
Vineland-3	1196
Autism Spectrum Rating Scale (ASRS)	1095
Early Childhood Service Intensity (ECSII)	1008
Austim Diag. Observ. Schedule 2 (ADOS2)	916
Achenbach Child Beh Checklist (CBCL)	444
Mullen Scales of Early Learning	397
Wechsler Intell Scale for Child. (WISC-V)	369
Achenbach-Parent/Caregiver Rpt (C-TRF)	357
Behavior Assessment System for Children3	318
Conners 3—Parent Report	279
Behavior Rating Inventory of Exec Funct	253
Sensory Profile—Short	235
Multi. Anxiety Scale for Children (MASC2)	233
Childhood Autism Rating Scale-2 (CARS2)	224
Wechsler Pre. Scale of Intell. (WPPSI-IV)	144
Social Language Development Test	124
Childhood Depression Inventory-2 (CDI2)	122
Disruptive Behavior Rating Scale	115
Differential Abilities Scales (DAS-II)	103

Test information for tests used for more than 100 clients; only SDQ analyzed in regression models

For ongoing clients with existing mental health diagnoses, annual diagnostic assessments were completed by mental health professionals during a 1 h interview that included an observation of the parent with the child. These may have included updated testing as part of a separate appointment as needed. The sample consisted of n = 3402 (44.2%) Annual Assessments, n = 4286 (55.7%) Evaluations, and n = 7 (0.1%) Neuropsychological Assessments. Individual client information was pooled across assessment occasions.

Diagnostic interviews for both types of assessments are guided by a formatted document in the Electronic Medical Record system (EMR) that requires the clinician to query health, demographic, educational, and treatment history. The EMR system requires that all blanks be filled in by the clinician in order to complete the assessment document. One of the primary dependent measures in this study was the clinicians’ responses to a question from the EMR document “Trauma Reported”. If a clinician responded “Yes” to this question, several dropdown boxes are presented by the system to further categorize the events experienced (e.g. “Car accident”, “Domestic violence/abuse”, “Emotional abuse”, “Fire”). A text box is also available for clinicians to describe trauma in free text form. We have conceptualized these trauma types as PTE’s for the purposes of these analyses because it is unknown whether the child shows a traumatic stress reaction to the event. These options were dichotomized into a present/absent variable for the dependent measure used in the trauma report regression.

**Fig. 1:**
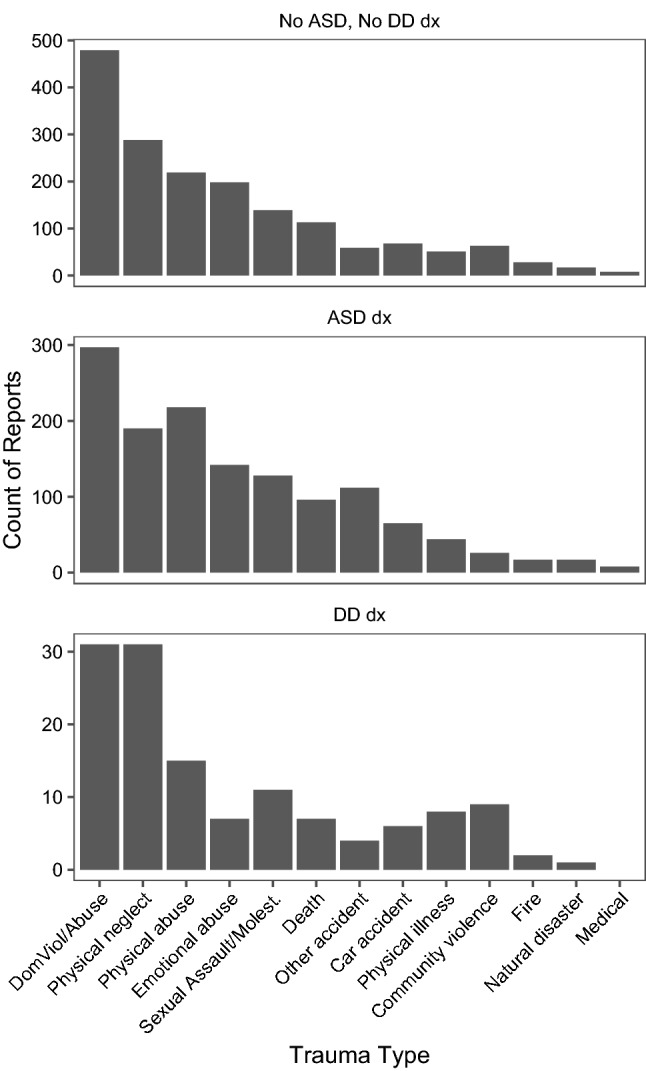
Types of trauma reported, by neurodevelopmental diagnostic group. *ASD* autism spectrum disorder diagnosis, *DD* developmental disability. Removed Other, None Reported categories

See Fig. 1 for commonly reported types of trauma by diagnostic group.

Diagnostic information was extracted from the EMR system and included DSM-IV (American Psychiatric Association 1994), 5 (American Psychiatric Association 2013), and ICD 9 (World Health Organization 1980), and 10 (World Health Organization 1993) systems of classification. Diagnostic data was combined across all evaluations to determine whether children had ever been diagnosed with any ASD (ICD codes 299.x, DSM codes: F84.0, 84.3,84.5, 84.8) or Intellectual/Developmental Disorders (ICD 315.9, 317,318,319, DSM codes: F70, 71, 72, 73, 78, 79). The dependent measure used in the second logistic regression were the types of trauma related diagnoses provided to the child at the conclusion of the assessment. These were grouped into levels and ordered from least to most common. The most common category was “no trauma, stressor-related diagnoses” which served as the lowest level of the categorical variable. The second level included the DSM-5 diagnoses: reactive attachment, disinhibited social engagement disorder, acute stress disorder, adjustment disorders (excluding unspecified adjustment disorder), and other specified or unspecified trauma and stressor-related disorders (ICD codes: 313.89, 308.3, 313.89, 309.89, 309.9; DSM codes: F94.1, F43.0, F94.2, F43.8, F43.9). As with other categorical variables in the regression model, the least common values in the data set was used as the highest level of the trauma outcome variable, in this case, the diagnosis of PTSD (ICD code 309.81; DSM code: F43.10) was the least common. The diagnosis Unspecified Adjustment Disorder was used administratively in billing for some clients and was excluded from the analysis.

Negative life events were also collected in the unstructured interview and recorded in the EMR system. This question was recorded as “stressors” and was classified by the clinicians using check boxes and included an “Other” category with a free text blank to use for further description. Clinicians also queried the number and types of living situations (e.g. foster home, relative’s home, homeless shelter) using a similar classification system and these were considered as another potential NLE.

### Participants

The sample was predominantly male (77.4%) which is typical of clinics seeing a high proportion of clients for Autism assessments. The male to female sex ratio in this sample for children with ASD was 4.36:1, which is typical of samples of children with ASD. The DD sex ratio (2.28:1) was similar to that of the comparison group of children with other mental health disorders (2.25:1). Clients who self-described using the race, “White” accounted for 23% of the sample. Ninety-one percent of families described the primary language of their child as being English, and 7.9% of clients served at the provider overall used interpreters for at least one session. Income and socio economic status data were not collected by the provider.

During the course of the study period, the provider began using online scoring for the Child Behavior Checklist (CBCL; Achenbach 1994), and Vineland Adaptive Behavior Scales-3, (VABS, Sparrow et al. 2016) assessments which allowed matching by names and dates of birth for a subset of participants in the study. These were available for too few participants to use in regression models but are described here to help describe the study. For the comparison mental health group VABS ABC standard scores showed a median of 75 (SD = 15.65), while those with ASD showed a median of 71(SD = 14.52). On the domain scores of the VABS, children in the comparison group had median scores of 78 (SD = 17.22) for Communication, 77 (17.80) for Daily Living, and 76 (16.56) for Social domain, while those in the ASD group had median scores of 71 (21.58) for Communication, 74 (16.74) for Daily Living, 68 (14.73) for Socialization domains. VABS data was available from too few participants with DDs (N < 10) to provide estimates of adaptive behavior. The CBCL Total Problems scale showed the greatest differences between groups. On this scale the DD group had median Total Problems score of 83 (SD = 27.41); the ASD group had median Total Problems score of 60 (28.88); the comparison mental health disorders group had a median total problems score of 63 (29.05).

### Instruments

Data was exported from the EMR system and cleaned, and descriptive statistics created using the R software (R Core Team 2018) using packages including data.table (Dowle and Srinivasan 2018) and tidyverse (Wickham 2017) for data cleaning and joining. Regression models were computed using base R packages (R Core Team 2018) and the package MASS (Venables and Ripley 2002). The package ggplot2 (Wickham 2016, p. 2) was used for graphing. Plots of regression effects were created using SJplot (Lüdecke 2018).

Use of the Strengths and Difficulties Scale (Goodman 2001) was mandated by state regulations at the beginning of services and at 6 month intervals for all clients. This scale was completed by caregivers and was scored and entered into the EMR system. The highest reported score of all scores on file per individual were used in the analyses. These scores were converted to categories (Close to average, Slightly raised, High, Very high) using the four band solution cut scores provided in the scoring instructions (“Scoring the SDQ,” 2019). For example, for Total Score, band cuts for the 4 to 17 year old form of the SDQ correspond to scores as follows: Close to average = 0–13, Slightly Raised = 14–16, High = 17–19, Very High = 20–40. See Table 2 for a summary of raw SDQ scores by group.

**Table 2:** SDQ median scores by trauma diagnosis and group

Group	Trauma diagnosis	Conduct	Emotional	Hyper- activity	Peer Problems	Prosocial	Total score
MH comparison	None	4 (2.53)	5 (2.74)	8 (2.54)	4 (2.28)	7 (2.30)	20 (6.63)
MH comparison	PTSD	5 (2.56)	5 (2.69)	8 (2.46)	4 (2.35)	7 (2.28)	22 (6.63)
MH comparison	RAD/stress	6 (2.04)	4 (2.65)	9 (2.49)	5 (2.25)	7 (2.50)	23.5 (6.90)
ASD	None	4 (2.32)	4 (2.65)	8 (2.20)	6 (2.07)	6 (2.43)	21 (6.06)
ASD	PTSD	5 (2.46)	6(2.51)	9 (2.06)	6 (2.00)	6 (2.29)	24 (5.65)
ASD	RAD/stress	6 (1.86)	6 (2.98)	9 (1.30)	6 (2.37)	5 (2.56)	26 (5.48)
DD	None	4 (2.64)	4 (2.59)	8 (2.34)	5 (2.31)	6 (2.45)	20 (6.86)
DD	PTSD	7 (2.59)	5 (2.16)	10 (2.46)	6 (1.85)	7 (2.45)	25 (6.49)
DD	RAD/stress	8 (1.37)	4.5 (3.74)	10 (0.84)	5 (1.17)	4.5 (2.07)	25.5 (3.97)

Median (Standard Deviation) scores by trauma diagnosis and group. Strengths and Difficulties Scale (Goodman 2001)

*MH Comparison* group of clients with other Mental Health diagnoses, *PTSD* post traumatic stress disorder, *RAD* reactive attachment disorder, *Stress* acute stress disorder

### Data Analysis

Data from multiple assessments were reduced to a single value for each variable per participant. To maximize the variability in the sample, categorical variables (such as living situations and types of trauma) from multiple reports were reduced to retain the value that was least prevalent in the overall sample. This strategy was chosen because rare events may be associated with trauma, and because one of the aims of the research is to discover potential events and types of situations that might serve as indicators of trauma for future screening. By retaining rare events, the opportunity to discover these indicators is maximized. Categories of variables present in less than 0.1% of assessments were combined to create the “Other” category. Any participant who did not respond to a question was given a value of “Not Disclosed”; this value was analyzed within the regression models. No data was missing for reasons other than non-disclosure from participants. Diagnostic variables included any diagnosis within a category (e.g. Autism) at any time; diagnoses were reduced to three groups ASD, DD, and No ASD or DD. Trauma/Stressor related diagnoses were similarly reduced to three levels: (1) no trauma related diagnosis, (2) reactive attachment, disinhibited social engagement, acute stress, adjustment disorders, or other trauma and stressor-related diagnoses, and (3) post-traumatic stress disorder (PTSD). These were considered as an ordered variable from least to most common as an outcome variable for the multinomial ordinal logistic regression. Results of the regression models are displayed and presented using odds ratios, which describe the relative likelihood of an outcome compared to the most common level of the predictor. These were: Race = White, Primary Language = English, No ASD/DD, Living situation = ”None reported”, Trauma type = ”None”.

SDQ categorical scores were treated as an ordinal variable and entered into regression models as linear, cubic, and quartic predictors. The linear predictors are described in the results below as they provided the best overall model fit based on Akaike Information Criteria (Akaike 1974) values.

Number of living situations was created by counting the number of distinct living situations reported from all evaluations and diagnostic assessments for each client. The number of stressors was created by counting the distinct stressors reported in assessment forms for an individual across assessment occasions.

## Results

### Trauma Reports

#### Trauma Exposure

Trauma was reported in 50.6% (n = 1261) of clients in the comparison group, 40.3% (n = 95) of the children with DD, and 23.5% of children with ASD (n = 1166). Logistic regression showed that trauma reports were less likely in children with diagnoses of ASD or DD when controlling for the predictors in the model. Compared to children with mental health diagnoses, children with ASD had an odds ratio (OR) of .34 (SE = 0.07, t = − 16.1, p < 0.001, CI [0.48, 0.91]), in other words they were 66% less likely (1-OR) to report trauma than the comparison group (children with other mental health diagnoses). Children with DD diagnoses were slightly more likely to report trauma than those with ASD but still were below the level of those in the other mental health disorders group (OR  0.66, SE = 0.16, t = − 2.57, p < 0.05, CI [0.48, 0.91]).

#### Living Situations

The groups differed in the proportion of participants who lived in two or more living situations. The comparison group reported 21.0% living in two or more living situations (n = 524), those with reported DD 17.4% (n = 41), and those with ASD reported 10.2% (n = 505). The largest increase in probability of reporting of trauma was associated with the number of living situations reported. For each increase of one distinct living situation reported, the likelihood of reporting trauma went up by 2.41 times (SE = 0.10, t = 8.41, p < 0.001, CI [1.97, 2.96]). The specific situation most associated with trauma reporting was foster care placement (OR 11.46, SE = 0.32, t = 7.69, p < 0.001, CI = [6.16, 21.33]). Other key living situations included living with relatives (OR 2.42, SE = 0.13, t = 6.9, p < 0.001, CI [1.88, 3.12]), living in homeless shelters or orphanages (OR 2.28, SE = 0.23, t = 3.63, p < 0.001, CI [1.46, 3.56]), or living in adoptive homes (OR 3, SE = 0.21, t = 5.3, p < 0.001, CI [2, 4.5]).

#### Negative Life Experiences (NLEs)

The number of participants per group who experienced at least one NLE was relatively equal across groups. In the comparison group of children with other mental health diagnoses, 78.9% experienced at least one NLE (n = 1968). In the DD (n = 186) and ASD (n = 3744) groups, 78.8% and 75.4% reported at least one NLE. Negative life events were also highly associated with risk of trauma report. Each one distinct NLE was associated with a 1.18 risk increase in reporting trauma (SE = 0.02, t = 7.81, p < 0.001, CI [1.13, 1.22]). All tested NLEs predicted increases in trauma reporting except financial stress, employment, lack of support, and limited English. The most important NLEs were: Legal issues (OR 4.43, SE = 0.2, t = 7.4, p < 0.001, CI [2.98,6.56]), custody conflicts (OR 4.05, SE = 0.19, t = 7.34, p < 0.001, CI [2.79,5.89]), family conflicts (OR 3.2, SE = 0.15, t = 7.59, p < 0.001, CI [2.37, 4.32]), divorces or separations (OR = 2.38, SE = 0.16, t = 5.51, p < 0.001, CI [1.75, 3.23]), deaths (OR 2.20, SE = 0.14, t = 5.44, p < 0.001, CI [1.75, 2.92]), family medical issues (OR 2.11, SE = 0.15, t = 4.91, p < 0.001, CI [1.57, 2.84]), family mental health issues (OR 2.04, SE = 0.17, t = 4.14, p < 0.001, CI [1.46, 2.86]), and transportation issues (OR 1.86, SE = 0.19, t = 3.20, p < 0.01, CI [1.27, 2.73]).

#### Demographic Variables

Having a non-disclosed or uncategorized race was associated with lower levels of trauma report than White participants (OR 0.81, SE = 0.07, t = − 3.02, p < 0.01, CI [0.71, 0.93]). Non-English speaking participants did not differ from English speaking participants. Age in years was associated with an increase in reporting trauma with each year increase in age associated with a 1.08 increase in trauma report odds (SE = 0.01, t = 9.92, p < 0.001, CI [1.07, 1.10]). Sex was not associated with differing levels of trauma reports.

#### SDQ Scales

Increases in the SDQ Conduct Problems subscale classification (e.g. from “close to average” to “slightly elevated”) resulted in a 1.58 fold increase in trauma reporting (SE = 0.06, t = 7.27, p < 0.001, CI [1.39, 1.78]). No other scales of the SDQ showed a linear relationship with trauma report.

**Fig. 2:**
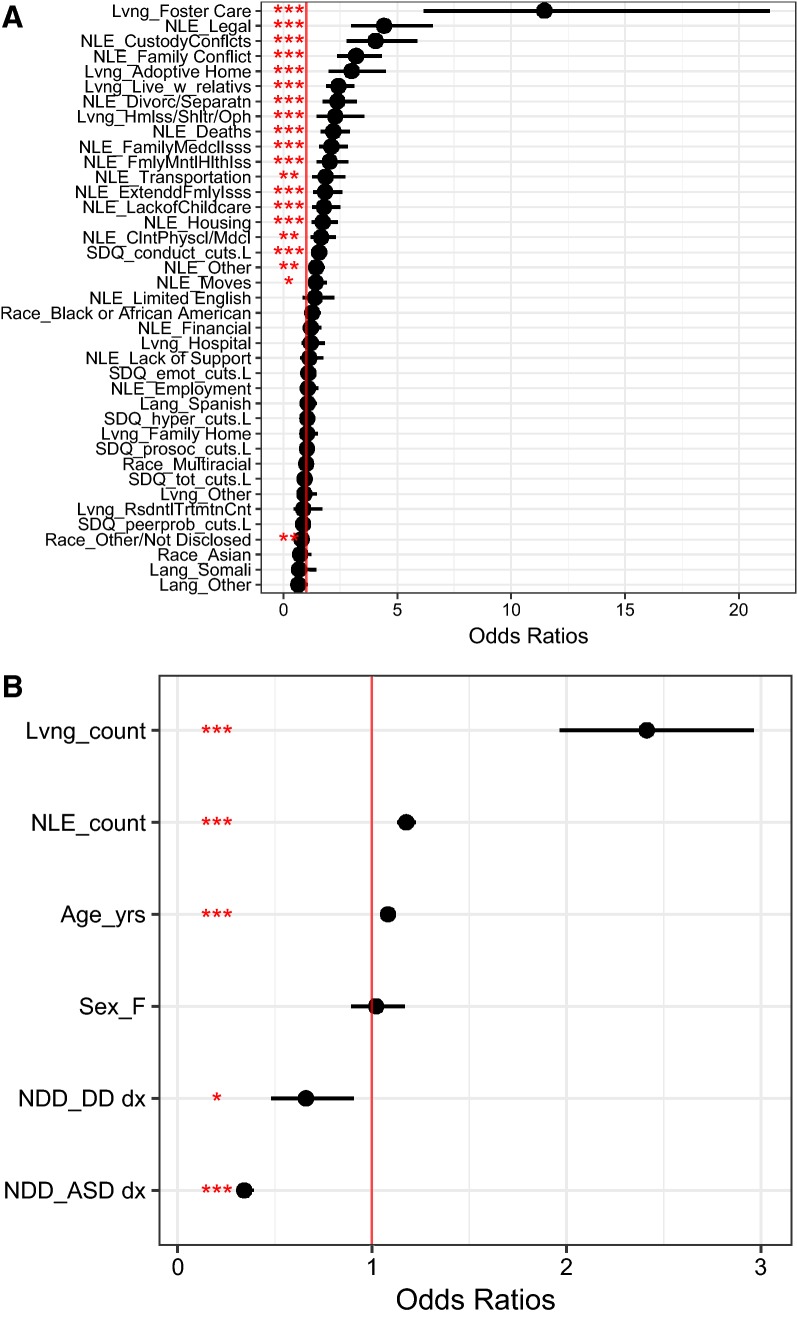
Trauma report logistic regression effects. **a** (top) Predictor Variables with more than three values (NLEss, living situations, race, language). **b** (bottom) Predictor with three levels and quantitative predictors. Vertical red line indicates no change in probability compared to comparison levels of NDD = ASD, Lang = English, Sex = M, Race = White, ACE = None Reported, Lvng = Not Reported. Abbreviations used: *p < .05, **p < .01, ***p < .001, *Lvng* living situation, *NLE* negative life events, *F* female, *NDD* neurodevelopmental disorders, *DD* developmental disabilities, *Lang* language, *RsdntlTrtmtnCnt* Residential Treatment Center, *Hmlss/Shltr/Oph* homeless/shelter/orphanage, *Live_w_relativs* live with relatives, *FamilyMedclIsss* family medical issues, *FmlyMntlHlthIss* family mental health issues, *ExtenddFmlyIss*s extended family issues, *ClntPhyscl/MdcI* client physical/medical issue, *Divorc/Separatn* divorce/separation. *SDQ* strengths and difficulties questionnaire (SDQ; Goodman 2001). *L* linear effect of ordinal variable

See Fig. 2 for odds ratios and confidence intervals for both qualitative and quantitative variables.

### Trauma Related Diagnoses

#### ASD and DD Diagnoses

Similar to the trauma report regression, a significant difference was found in trauma diagnoses for children with ASD and DD. In the diagnostic regression those with ASD showed the lowest likelihood of trauma diagnosis (OR 0.15, SE = 0.09, t = − 21.2, p < 0.001, CI [0.12, 0.17]) and those with DD (OR 0.57, SE = 0.19, t = − 2.93, p < 0.01, CI [0.39, 0.83]) being slightly more likely to be diagnosed with a trauma or stressor-related diagnosis than those with ASD, but still significantly less likely to be diagnosed with a trauma related diagnosis than children with other mental health diagnoses. In terms of specific diagnosis categories, 29.81% of children in the mental health comparison group had a PTSD diagnosis, compared to 14.83% of the group of children with DDs, and 4.25% of children with ASD. Reactive Attachment Disorders or Acute Stress Disorders were seen for 1.73% of the children in the comparison group, 2.54% of the children in the group with DDs, and 0.34% of the group with ASD.

#### Living Situations and Negative Life Experiences

Also similar to the results for trauma reporting, trauma-related diagnoses were associated with increases number of living situations (OR 1.62, SE = 0.08, t = 6.06, p < 0.001, CI [1.38, 1.89]), with the experience of foster care being a key predictor as in the previous trauma report regression model (OR 2.9, SE = 0.21, t = 4.98, p < 0.001, CI [1.91, 4.42]). Other key living situations that were associated with trauma diagnoses were living in an adoptive home (OR 2.81, SE = 0.2, t = 5.21, p < 0.001, CI [1.91, 4.15]), living with relatives (OR 1.96, SE = 0.17, t = 3.99, p < 0.001, CI [1.41, 2.73]), and living in a homeless shelter or orphanage (OR 2.03, SE = 0.22, t = 3.27, p < 0.01, CI [1.33, 3.11]). These findings also corresponded with the trauma report model.

The number of distinct NLEs were predictive of increased levels of trauma diagnosis (OR 1.07, SE = 0.03, t = 2.63, p < 0.01, CI [1.02, 1.12]). Specific NLEs were also associated with increased risk of trauma diagnosis; Family mental health issues (OR 2.85, SE = 0.2, t = 5.22, p < 0.001, CI [1.92, 4.22]) legal issues (OR 1.44, SE = 0.18, t = 1.99, p < 0.05, CI [1.01, 2.05]), custody conflicts (OR 2.18, SE = 0.22, t = 3.5, p < 0.001, CI [1.41, 3.38]), and family conflicts (OR 2.3, SE = 0.19, t = 4.31, p < 0.001, CI [1.57, 3.36]) were associated with increases in trauma diagnosis. These same predictors were important in the trauma report regression.

#### Demographic Variables

Also similar to the trauma report results, having Other/Not Disclosed race (OR 0.55, SE = 0.09, t = − 6.41, p < 0.001, CI [0.46, 0.66]) was associated with reduced risk. In addition, in the trauma diagnosis model, being multi-racial was associated with slightly lower rates and intensity of trauma diagnosis (OR 0.72, SE = 0.17, t = − 2, p < 0.05, CI [0.52, 0.99]). While language was not a significant predictor of trauma reports in the previous regression model, speaking other non-English languages (OR 0.25, SE = 0.61, t = − 2.3, p < 0.05, CI [0.07, 0.81]) was associated with reduced risk for trauma diagnoses albeit with a small effect size.

#### SDQ Scales

As in the trauma report regression model, the Conduct Problems subscale was associated with increased likelihood of trauma-related diagnoses (OR 1.78, SE = 0.09, t = 6.42, p < 0.001, CI [1.49, 2.12]). Unlike the trauma report regression model, the diagnosis model showed that higher SDQ Hyperactivity (OR 0.82, SE = 0.1, t = − 1.96, p < 0.05, CI [0.67, 1]) was associated with a slightly lower level of trauma diagnosis.

**Fig. 3:**
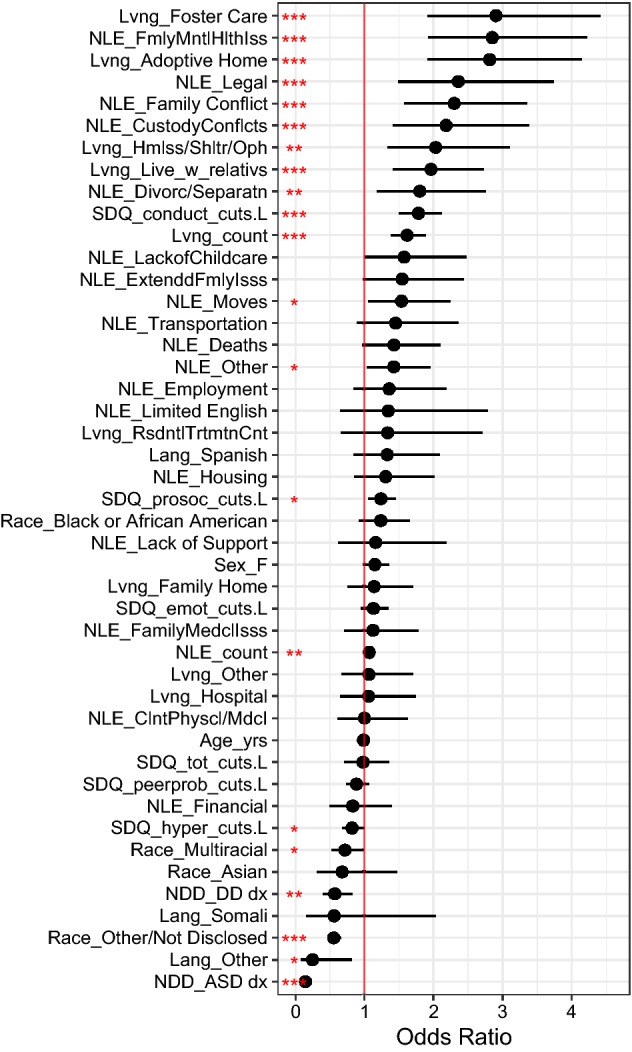
Trauma and stressor related diagnoses multinomial regression effects. *Lvng* living situation, *NLE* negative life events, *ASD* autism spectrum disorders, *DD* developmental delays/disabilities. Red vertical line = level of probability for comparison values/groups

See Fig. 3 for the plot of effects.

## Discussion

This study examined predictors of trauma reports and trauma/stressor-related diagnosis in a large sample (N = 7695) of clients at a specialty mental health provider for children and adolescents. The presence of qualitative descriptors of negative life events, demographic information, and standardized data from parent rating scales from a large sample allowed for the assessment of the relative contribution of each of these to predicting trauma exposure and trauma related diagnoses in children with ASD, DD, and those with other mental health disorders. As hypothesized, NLEs, including quantitative NLE’s such as the number of living situations, and the number of negative life events, as well as key categories of NLEs, namely legal issues, family conflict, custody and family conflicts, divorce/separations, family mental health issues, and deaths were predictive of trauma report and trauma related diagnoses across groups compared in the study. Regardless of diagnostic group, these variables served as risk factors.

Although the same predictors were associated with both trauma reporting and trauma related diagnoses across children with ASD, those with DD, and the comparison group of children with other mental health disorders, we found lower rates of both trauma reporting and trauma related diagnoses for the DD and ASD groups. Even while controlling for sex, age, SDQ scores, and number and types of negative life events and living situations, the ASD and DD groups were significantly less likely to have caregiver reported trauma exposure and lower levels of clinician diagnosed trauma related diagnoses compared to the children without ASD and DDs. This may be due to difficulties in seeing symptoms of trauma against the background of ASD or DD symptoms, or could be due to differences in parent reporting.

We found that regardless of group, NLEs served as risk factors. Quantitative NLE’s such as the number of living situations and the number of negative life events, as well as key categories of NLEs, namely legal issues, family conflict, custody and family conflicts, divorce/separations, family mental health issues, and deaths were strongly associated with both trauma diagnoses and with trauma reports. These variables might provide a target for future trauma screening efforts that is predictive across children receiving services within community mental health settings. Within the clinical diagnostic interviews, discussion of the number and types of living situations, along with other life experiences (e.g., divorce), may provide less invasive topics that could lead to a further discussion of trauma. Results also suggest that the Conduct Problems score from the SDQ may also be worthwhile to include in screening efforts. Future work will examine prospective use of these indicators to flag clients at high risk to use more comprehensive trauma assessment tools. Because the predictors were entered together into the models, these associations are important when taking into account ASD and DD diagnostic categories and other quantitative variables (e.g. SDQ scores) and categorical variables such as age and sex.

Controlling for NLE’s, SDQ scores, and demographic variables, the ASD and DD groups were less likely to have caregiver reported PTEs. This finding is in contrast to other studies which have reported higher PTE’s in ASD and DD groups compared to typically-developing children (Berg et al. 2016; Hall-Lande et al. 2015; Spencer et al. 2005; Sullivan and Knutson 2000). Prevalence rates of PTE exposure in our study were found to be 24% for the ASD group, 40% for the DD group, and 51% for the comparison mental health disorders group. Although low, the prevalence rate of exposure to PTEs for the ASD group (24%) was somewhat similar to what has been reported by caregivers within inpatient and outpatient mental health settings in the United States. For example, within community mental health clinics, Mandell et al. (2005) found caregiver-reported exposure to physical and sexual abuse at approximately 30% in a sample of children with a mean age of 11 years. Within inpatient hospital settings, the prevalence of caregiver-reported exposure to physical, sexual, and/or emotional abuse was found to be similar, at 28% for individuals with ASD aged 4–21 years (Brenner et al. 2018). What these studies have in common is the reliance on caregiver report on varying categories of trauma exposure within clinical settings. It could be the case that caregivers under-reported trauma exposure. Indeed, Hall-Lande et al. (2015) used administrative child maltreatment records within the Child Protection System and found that children with ASD and DD had higher rates of CPS involvement than typically-developing children. It could also be the case that clinicians and/or caregivers are more likely to consider limited categories of trauma exposure (e.g. physical abuse) which might miss other PTEs in the child’s life. Studies that use a broader criteria for trauma exposure that include stressors across contexts and witnessing trauma more closely approximate the categories used in the current study and these studies have documented higher trauma prevalence rates. For example, rates between 55 and 60% have been observed in children 3–17 years old been exposed to a potentially traumatic event (Berg et al. 2016; Taylor and Gotham 2016). Similarly, Taylor and Gotham (2016) reported that 60% of high school students with ASD reported exposure to PTEs. They also found evidence that stressors common within family and peer contexts such as divorce and bullying, were perceived as traumatic by youth with ASD. This body of literature suggests that interview methods, settings discussed, and types of events queried may strongly impact the ability to detect traumatic events. Capturing the full range of traumatic experiences for those with disabilities will entail careful consideration of the types of screening questions, and may require data gathering across multiple sources.

Another explanation for the low levels of trauma exposure in the ASD and DD groups is that trauma exposure was under-reported by caregivers because they were unaware of the traumatic event. Differences in social cognition could impede those with ASD and DD from understanding the social context of PTEs, resulting in less trauma reporting to caregivers. Indeed, Loveland et al. (2001) reported that youth with ASD had more difficulty identifying verbal inappropriateness (i.e., insults) in video scenes of social interactions, than typically-developing peers. Besides differences in perceiving social situations, language and communication difficulties, which are seen in up to 63% of children with ASD (Levy et al. 2010), could impede people with autism from reporting PTEs to caregivers and mental health professionals and might account for the differences between children with ASD and those with DD diagnoses in this study. Further research is needed examining how factors such as language, social, and cognitive functioning may impact trauma exposure and the child’s ability to understand and report traumatic experiences. The effects of language, social, or cognitive functioning on our results is unknown as testing data was not available to analyze.

Considering that group differences were seen in both PTE exposure reports and in trauma related diagnoses, both child and parent specific reporting barriers and diagnostic factors may play a role. The lower rates of diagnosis of trauma related disorders in this study could be due to symptom overlap between ASD, DD, and trauma related diagnoses. Focusing on only the diagnostic category of PTSD, for example, our study found lower rates of diagnosis the ASD (at 4.25%) and DD (at 14.83%) groups. This fits within a wide range of estimates seen in the literature which come from a wide range of care settings, such as an inpatient hospital (Brenner et al. 2018; Mehtar and Mukaddes 2011). Diagnostic overlap within categories may account for some of the difficulties in diagnosing the effects of trauma apart from the symptoms of ASD and DD. For example, intrusive recurring thoughts that are characteristic of PTSD may be interpreted as restricted or fixated interests seen in ASD (American Psychiatric Association 2013). Night terrors or other sleep disturbances that are characteristic of PTSD might be interpreted as insomnia which is common in ASD, affecting 40–80% of those with ASD (Cortesi, et al. 2010). Avoidance of seeking comfort and lack of social and emotional responsiveness, symptoms of Reactive Attachment Disorder, are also seen in ASD (American Psychiatric Association 2013).

Research suggests classes of behaviors associated with PTE exposure in people with ASD which might provide ways to disentangle symptom overlap to improve the gaps in diagnoses found in the current study. Increased levels of lethargy and irritability have been found (Brenner et al. 2018), along with aggression, distractibility, and appetite disturbances in the ASD groups with reported trauma compared to no-trauma ASD groups (Mehtar and Mukkades 2011). Similar findings have been noted in adults with DDs with trauma histories (Ryan 1994). In children with ASD and sexual abuse histories, higher levels of sexual acting out and sexually assaultive behavior, suicide attempts, and running away from home were seen compared to the non-abused group (Mandell et al. 2005). Other studies have noted no declines in pre-existing adaptive functioning in children with ASD with reported trauma exposure nor changes in core ASD symptomatology (Brenner et al. 2018) while one study has reported deterioration in skills (Valenti et al. 2012). Valenti et al. (2012) examined adaptive behaviors of children with ASD attending an autism day school in Italy, some of whom had been exposed to a severe earthquake. Six months following the earthquake, those with trauma exposure showed significant declines in communication, socialization, daily living, and motor skills as compared to the non-exposed group. At the 1-year follow-up, children in the exposed group had made improvement in some areas but were still below baseline levels. Taken together, there is evidence to suggest that trauma exposure in those with ASD may lead to a deterioration in social, language, and adaptive functioning, and the presentation of new problems. However, a clear clinical picture of trauma-related effects in those with a disability compared to those without has not emerged which may contribute to the under-detection and under-diagnosis of trauma-related disorders in this population.

Another source of the differences between groups on trauma related diagnoses could be the phenomenon of diagnostic overshadowing (Reiss et al. 1982). This phenomenon is well documented and describes ways clinicians and caregivers might attribute behaviors, a worsening of behavior, or a regression of skills as being part of a person’s primary diagnosis rather than being due to other factors. Brenner et al. (2018) compared children within an inpatient hospital with maltreatment histories to those with no abuse history on parent-reported PTSD symptoms, controlling for age, nonverbal IQ, and verbal ability. Of the 10 PTSD symptoms examined, the group with maltreatment history only showed higher levels on three symptoms: distressing memories, and intrusive thoughts from DSM-5 diagnostic criteria B, and loss of interest from DSM-5 diagnostic criteria D. Both the abused and non-abused groups showed similar levels of irritability, avoidance, anger, fear, temper tantrums, sleep disturbances, and attention problems. This illustrates some of the complexity involved in detecting PTSD symptoms against the backdrop of ASD.

### Limitations

The clinical data that forms the source data for these analyses are both a strength and limitation of the study. In this study, assessment of trauma and stressor-related diagnoses were not conducted using a standardized assessment tool; clinicians used idiosyncratic interviewing methods and categorized trauma based on idiosyncratic methods. While this likely lead to variability in the outcome measures, it means that predictor effects are robust to a wide variation in clinical use. It also suggests that the findings may be applicable to other clinical settings.

Another primary limitation of the study is the high number of non-disclosed responses in demographic categories such as race (e.g. 59.5%). Because this is due to participant non-disclosure instead of recording error, it cannot be considered missing at random. The provider queries race, language and ethnicity during phone intakes while clients are requesting services and scheduling first appointments and this may partially account for the low response rate on this variable. Unmeasured variables or undisclosed variables such as socio-economic status might differ between the groups in this study and might provide an alternative explanation for differences in rates of diagnosis and reporting.

In this study, multiple assessments were collapsed to a single value per participant. Future work could implement time series modeling to determine whether predictors are more effective at different ages, or whether specific predictors appear prior to a report of trauma. Stratification of trauma related diagnoses into ordinal categories was done to better elucidate the varying levels of diagnoses seen in the sample across these diagnoses. Whether this ordering corresponds to increases in the severity of diagnosis is unknown. It is likely that, despite the long time scale of the data, some clients diagnosed in a less common category such as Acute Stress, may eventually “convert” their diagnosis to PTSD. A non-ordinal model was also run and showed a similar pattern of results, and slightly better fit to the data based on AIC scores, however, for ease of interpretation between the binomial and multinomial models, the ordinal model was retained. Reliability of clinician methods of querying trauma are unknown in this study, as the results and diagnoses provided by clinicians could not be compared to standardized assessments.

Outcomes of interest in this study were gathered from the caregiver(s) present at the child’s evaluation; thus, as is the case with single informant measures, reports of trauma exposure and trauma-related symptoms were subject to omission, bias, and misinformation. Future work should examine multiple informants (e.g., child, caregiver, teacher, child protection system), as there is evidence to suggest that in the case of trauma exposure, different types of trauma-related symptoms may be reported by different informants (Walrath et al. 2003). Our research does not allow conclusions to be made about the role of multiple reporters because while school reports and teacher questionnaires are used as part of the evaluations, caregivers were the only reporters that were always available for interview.

Beyond study-specific limitations, the field continues to lack large, population based, longitudinal studies that could describe the prevalence and incidence of trauma, adverse childhood experiences, PTSD, and other trauma and stressor-related diagnoses within ASD and DD populations. Because this study used existing data, we can describe what is associated with trauma reports or diagnoses, not what truly predicts these diagnoses.

### Future Work

In future work with this and similar clinical data sets, supervised machine learning based algorithms (e.g. random forest) could be applied to this data to obtain more distinct cut points from quantitative predictor variables, and ensembles of categorical variables to create screening indicators from this data. Because these approaches are robust to large numbers of predictor variables, additional variables might be tested using these approaches. Some of these methods also allow for time series elements within the data.

To better capture NLE’s for children with ASD and DDs, surveys such as the *Stress Survey Schedule for Persons with Autism and Other Developmental Disabilities* which was developed to assess for events that may be uniquely stressful to children with disabilities (Groden et al. 2001) may be helpful. Research using this questionnaire has found that children and adults with ASD view certain events (e.g., social demands, aversive sensory stimuli) as being more stressful as compared to typically-developing samples and those with an intellectual disability (Gillott and Standen 2007; Groden et al. 2001; Jansen et al. 2003). Notably, in the current study, the three groups identified similar rates of NLE’s. Further research should examine the use of tailored ratings that might provide insight into a broad array of NLE’s.

Further research is needed investigating the association between stressful experiences and trauma exposure in the development of psychopathology in those with a disability. This research will be critical to the development or modification of standardized trauma screening and assessment measures to be used with persons with disabilities. Adaptation and development of screeners for trauma exposures and trauma effects are also recommended for future studies. Several measures have been developed to measure trauma exposure and effects including PTSD in children with intellectual disabilities such as the Lancaster and Northgate Trauma Scales (LANTS; Wigham et al. 2014) and the Anxiety Disorders Interview Schedule- Children Intellectual Disabilities (ADIS-C-IDs; Mevissen et al. 2014, 2016). Currently it is unknown how screening or diagnostic measures may perform when applied to children diagnosed with ASD.

The current study suggests a high level of unidentified trauma exposure and under-diagnosis of trauma and stressor-related diagnoses in children with ASD compared to other groups of children with mental health and DD diagnoses. Future work should pilot brief screening tools to determine if rates of trauma exposure and diagnoses could be improved in ASD relative to other populations. There is evidence to suggest that trauma exposure in those with ASD may lead to a deterioration in social, language, and adaptive functioning, and the presentation of new problems. Longitudinal studies examining samples of children with ASD and DDs impacted by trauma are needed to better understand trauma effects against the backdrop of autism symptoms. A better understanding of the presentation of traumatic stress symptoms in children with ASD is critical for implementing appropriate intervention services for this population.

Finally, screening and diagnosis is only helpful in the presence of treatments. Many of the evidence-based interventions to treat the effects of trauma, such as Trauma-Focused Cognitive Behavioral Therapy, have been tested using randomized controlled trials among primarily typically-developing children and have shown moderate to high rates of improvement in mental health (de Arellano et al. 2014; Morina et al. 2016). Although general suggestions on how interventions can be adapted for use with disabled population have been made (Charlton et al. 2004), it is unclear whether these interventions would be as effective in disabled populations and how specific adaptations could be implemented to allow those with ASD and other disabilities to benefit in treatment. Research is needed examining the efficacy and effectiveness of these interventions using samples of children with disabilities, particularly those with impairments language, social, and cognitive functioning.
